# Soldier-Specific Modification of the Mandibular Motor Neurons in Termites

**DOI:** 10.1371/journal.pone.0002617

**Published:** 2008-07-09

**Authors:** Yuki Ishikawa, Hitoshi Aonuma, Toru Miura

**Affiliations:** 1 Graduate School of Environmental Science, Hokkaido University, Sapporo, Japan; 2 Research Institute for Electronic Science, Hokkaido University, Sapporo, Japan; Smithsonian Institution, United States of America

## Abstract

Social insects exhibit a variety of caste-specific behavioral tendencies that constitute the basis of division of labor within the colony. In termites, the soldier caste display distinctive defense behaviors, such as aggressively attacking enemies with well-developed mandibles, while the other castes retreat into the colony without exhibiting any aggressive response. It is thus likely that some form of soldier-specific neuronal modification exists in termites. In this study, the authors compared the brain (cerebral ganglion) and the suboesophageal ganglion (SOG) of soldiers and pseudergates (workers) in the damp-wood termite, *Hodotermopsis sjostedti*. The size of the SOG was significantly larger in soldiers than in pseudergates, but no difference in brain size was apparent between castes. Furthermore, mandibular nerves were thicker in soldiers than in pseudergates. Retrograde staining revealed that the somata sizes of the mandibular motor neurons (MdMNs) in soldiers were more than twice as large as those of pseudergates. The enlargement of MdMNs was also observed in individuals treated with a juvenile hormone analogue (JHA), indicating that MdMNs become enlarged in response to juvenile hormone (JH) action during soldier differentiation. This enlargement is likely to have two functions: a behavioral function in which soldier termites will be able to defend more effectively through relatively faster and stronger mandibular movements, and a developmental function that associates with the development of soldier-specific mandibular muscle morphogenesis in termite head. The soldier-specific enlargement of mandibular motor neurons was observed in all examined species in five termite families that have different mechanisms of defense, suggesting that such neuronal modification was already present in the common ancestor of termites and is significant for soldier function.

## Introduction

Social insects are characterized by highly organized social behavior, which is facilitated by an elaborate caste system. Colony members are differentiated into various castes, which all contribute to the fitness of the colony through the division of labor. Different castes exhibit a variety of morphological and behavioral characteristics that facilitate specific tasks in the division of labor [1]. In addition to immature individuals (larvae), termite colonies are composed of reproductive, worker and soldier castes. In instances when the colony is being threatened or damaged, such in the event of invasion by predators, workers escape deep into the colony and exhibit building behavior while soldiers employ specific defensive behaviors related to attacking their enemy aggressively [2]. Soldiers position themselves scattered about the nest, or they may patrol the nest surface antennating or attacking. There are numerous interspecific differences in the methods used by soldiers to attack; they may bite or “snap” with their mandibles, discharge defensive chemical secretions from their frontal glands, or plug the nest with their heads [2]. One common feature of all soldiers, however, is that they are characteristically aggressive and exhibit specific defense behaviors, which suggests that the specific control of these soldier-specific defense behaviors occurs in the nervous systems of soldiers.

Generally, the central nervous system (CNS) of insects consists of a brain (cerebral ganglion), a suboesophageal ganglion (SOG), three thoracic ganglia, and abdominal ganglia that are joined from the anterior to posterior of the body by two major connective nerves [3]. The brain plays an important role in integrating behavior, while the SOG controls the salivary ducts, certain muscles of the head-thorax junction, and the mouthparts, including mandibles [4], which are one of the distinguishing characteristics of soldier morphology.

Comparison of the CNS between castes provides important clues for understanding the types of nervous system specialization in social insects that are responsible for caste-specific behavior. Recent studies have demonstrated that certain regions of hymenopteran brains are modified and that this affects behavioral differentiation in these holometabolous insects [5]–[11]. It was found that the mushroom body changed according to the age or foraging experience of honeybees [5], [6]. The macroglomerulus of the antennal lobes are enlarged in large-bodied workers of the leaf-cutting ant, involved in the detection of the trail pheromone [7]. Other studies on hymenopterans suggest that the presence of the queen, mating experience, or age could also influence the concentration of biogenic amines or the expression of their receptors [8]–[11]. However, few studies have elucidated the mechanism underlying behavioral differentiation in hemimetabolous social insects such as termites, aphids, and thrips that have independently acquired eusociality [12]. To understand the general mechanisms underlying the elements of social behavior shared by social insects, increased knowledge of the nervous systems of hemimetabolous social insects is required. Caste-specific anatomical differences in size and allometry have been reported for the nervous systems of certain termite species [13], however, most of the information collected to date consists of fragmentary descriptions.

To further elaborate on soldier-specific modifications of the central nervous system, the authors undertook anatomical and histological examinations of the brains and SOGs of termites. The focal species was a damp-wood termite, *Hodotermopsis sjostedti* (Family Termopsidae; Order Isoptera), which have “biting-type” soldiers. This species was selected because they have large bodies and are thus well suited to anatomical examinations. In addition, “biting-type” soldier are considered to represent the evolutionarily ancestral condition [14]. After they hatch, larvae undergo six molts before becoming pseudergates, which function as workers. Pseudergates can subsequently differentiate into either soldiers or alates, although many remain pseudergates by repeating stationary molts [15]. Individuals that enter the alate line become nymphs first, before molting into alates, while individuals in the soldier line become soldiers through presoldiers [16], [17]. It has been widely reported that juvenile hormone (JH) plays an important role in termite soldier differentiation [18]–[20]. In *H. sjostedti*, soldier differentiation can be induced by applying a juvenile hormone analogue (JHA). To demonstrate how the CNS is modified during soldier determination, a method employing this hormonal control was used in this study. It was shown that there was a distinct nervous specialization accompanying the soldier differentiation in *H. sjostedti*, and that this phenomenon was common in all termite species so far examined.

## Results

### Morphometry of brain and SOG

**Figure 1:**
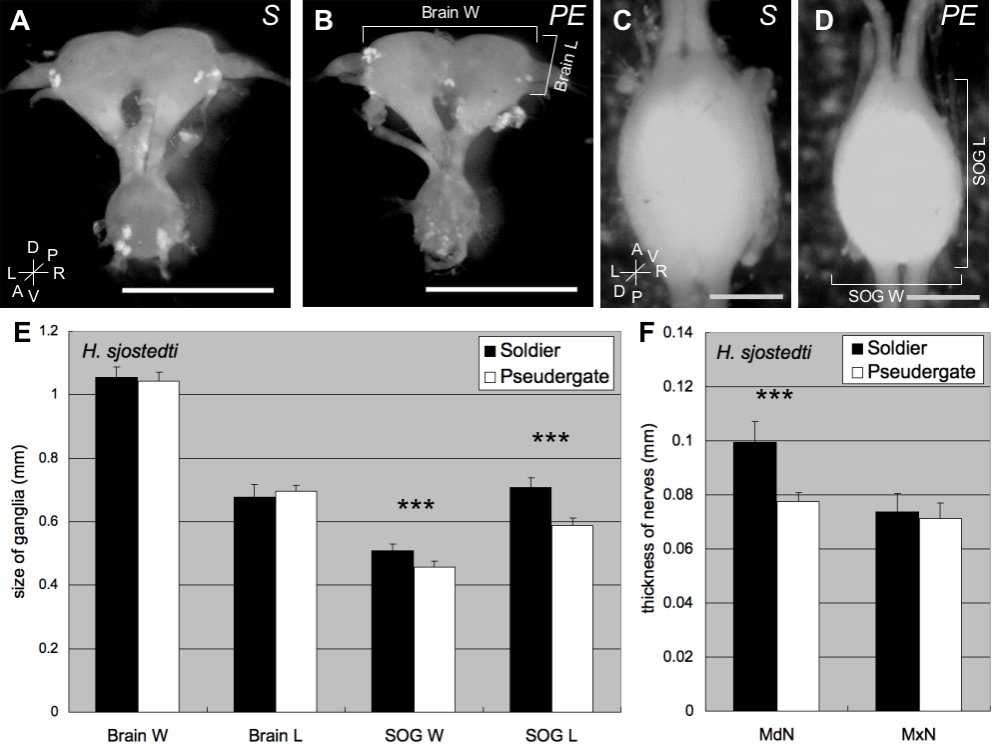
A comparison of size measurements for the brain and suboesophageal ganglion (SOG) of the cranial central nervous system (CNS) of soldier (*S*) and pseudergate (*PE*) castes in the damp-wood termite *Hodotermopsis sjostedti*. A, B: Anterior view of CNS from a soldier (A) and a pseudergate (B). C, D: Dorsal view of SOG from a soldier (C) and a pseudergate (D). Anterior end of SOG facing up (C, D). Scale bars indicate 1 mm (in A and B) and 0.5 mm (in C and D). e: The size comparison of brain width (Brain W), brain length (Brain L), suboesophageal ganglion width (SOG W), and suboesophageal ganglion length (SOG L) shown as a mean±SD (n = 10). F: Size comparison of the mandibular nerve (MdN) and maxillar nerve (MxN) shown as a mean±SD (n = 10). The size of the SOG from soldiers is larger than those from pseudergates. Moreover, the mandibular nerve of soldiers is thicker than it is in pseudergates. Asterisks indicate significant differences in the size between soldiers and pseudergates (Student's T-test: ***p<0.001).

While morphometric analyses showed that SOGs of the soldiers were significantly larger than those of the pseudergates (Length p<0.001, Width p<0.001; Student's-T test), no detectable difference in brain (cerebral ganglion) size was detected (Fig. 1A–E). In addition, measurements of mouthpart nerves innervated from the SOG revealed that, unlike maxillary nerves (MxNs), the mandibular nerves (MdNs) were thicker in soldiers than in pseudergates (Fig. 1F). We therefore expected for there to be some neuronal modification involved with the mandibular motor/sensory systems of soldiers.

### Retrograde tracing study using mandibular muscles in *H. sjostedti*

**Figure 2:**
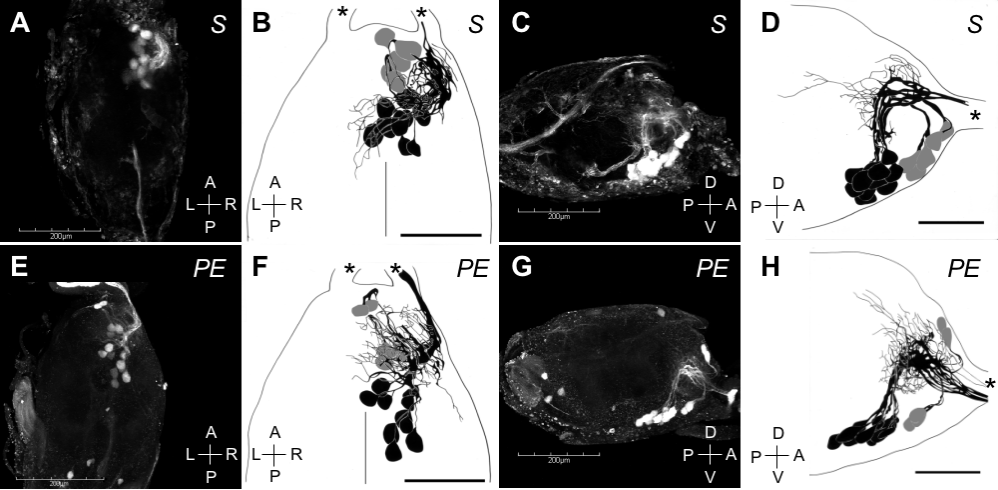
The mandibular motor neurons (MdMNs) stained by the retrograde tracing from the mandibular closer muscles of a soldier (A–D) and a pseudergate (E–H) in *H. sjostedti*. The somata of MdMNs are located in the anterior region of the SOG and constitute the anterior cluster which contained 5 neurons (colored in grey), and the posterior cluster which contained 12 neurons (colored in black). Confocal images (A, E) and schematic images (B, F) in the dorsal view are shown. Anterior end of SOG facing up (A, B, E, F). Confocal images (C, G) and schematic images (D, H) in the lateral view are also shown. Anterior is right (C, D, G, H). Scale bars in A, C, E and G show 200 µm and those in B, D, F and H show 100 µm. Asterisks indicate mandibular nerves.

Since the SOG is known to control the mouthparts and soldiers have markedly larger mandibles than pseudergates, we hypothesized that the large SOG in soldiers was related to the development of mandibular motor neurons (MdMNs). To prove this hypothesis, retrograde staining from mandibular closer muscles was performed. As a result, 17 MdMN somata and neurites were efficiently stained in the antero-ventral region (Fig. 2). Of these, five somata were located around the axon tract of the MdMNs, all of which showed variability with respect to position (anterior cluster; Fig. 2B, D, F, H, indicated in grey). The patterns of the neuronal branches were similar; moving backwards along their respective axons first before turning back on themselves to the mandibular nerve (MdN) together with remaining 12 neurons. The remaining 12 somata were stained in the ventro-median region of the anterior part of the ganglion, and were markedly less varied with respect to their positions (posterior cluster; Fig. 2B, D, F, H, colored in black). These axons extend to the dorsal region before turning orthogonally to the MdN. Most of the dendrites were restricted to the ipsilateral side, with only a few reaching the contralateral side. No significant difference was observed in the number of stained MdMNs between castes (data not shown).

**Figure 3:**
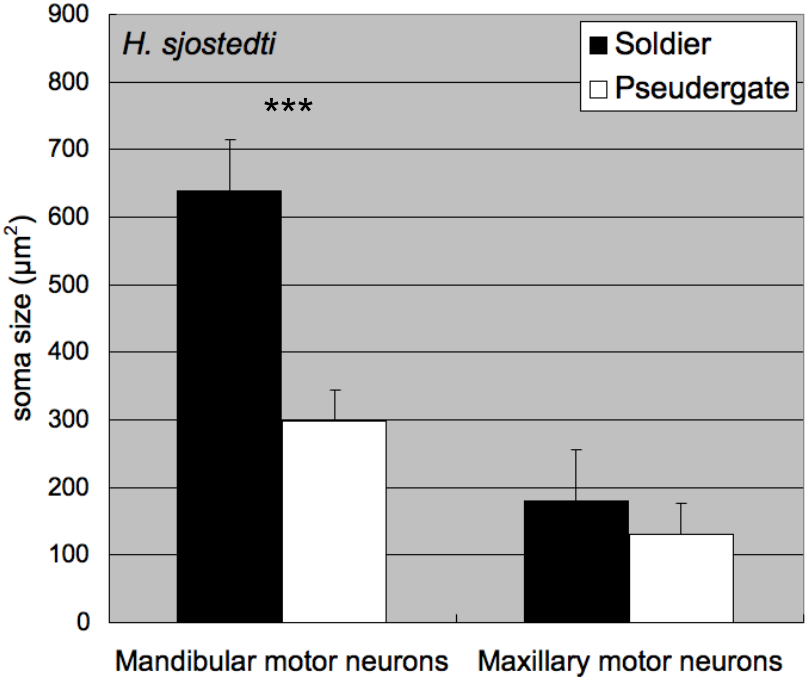
Size comparisons of MdMNs between soldiers and pseudergates in *H. sjostedti* (n = 5 for each caste). The areas of MdMN somata were measured and averaged, and MdMNs of soldiers were found to be twice as large as those of pseudergates. As opposed to the distinctive size difference in MdMNs (***p<0.001, Student-T test), the maxillary motor neurons show no significant difference in size (p>0.01, Student's T-test).

The size of the mandibular closer motor neurons appeared to differ between soldiers and pseudergates (Fig. 3). The average cross-sectional areas of the maximal MdMN somata sections in pseudergates and soldiers were 297.5±108.5 µm^2^ (mean±SD) and 638.3±168.0 µm^2^ (mean±SD), respectively. Consequently, the somatal size of the MdMNs in soldiers were approximately twice that observed in pseudergates, while those of the maxillary motor neurons showed no significant difference between castes (Fig. 3). The motor neurons innervating the mandibular opener muscles were also stained and were also found to be larger in soldiers than in pseudergates (data not shown). Hence, the soldier-specific enlargement of MdMNs was generally recognized. Similarly, while the corresponding neurons in alates (or imagos) were enlarged to some degree, they were smaller than those observed in soldiers (data not shown).

### Comparison of MdMNs during soldier differentiation

**Figure 4:**
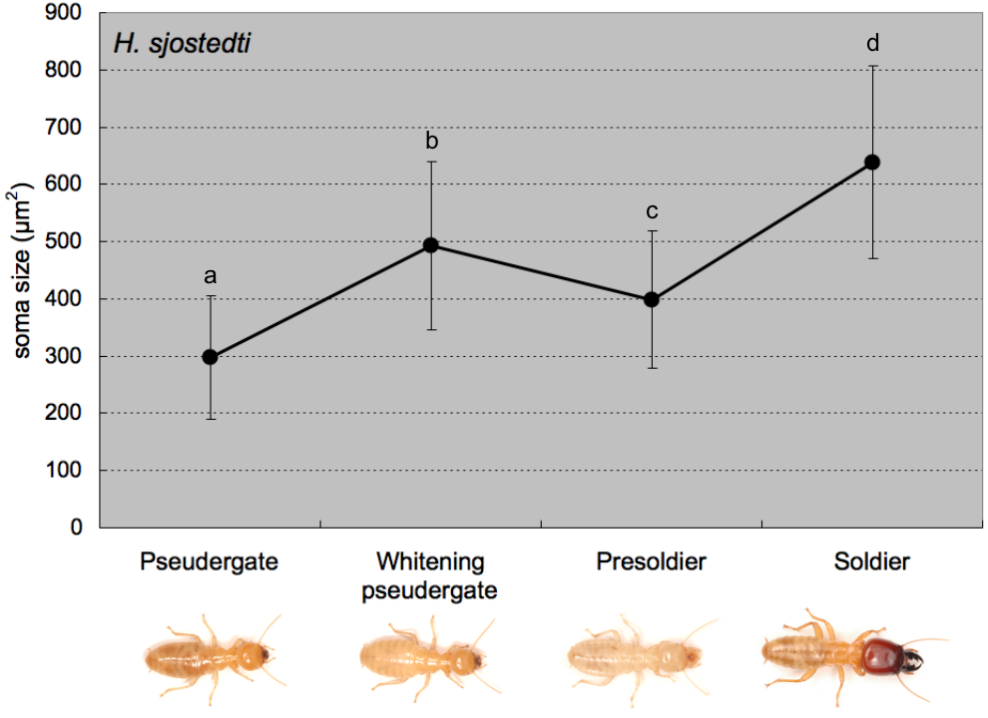
The transition of MdMN size during the course of soldier differentiation in *H. sjodtedti* (n = 5 for each caste). The areas of MdMN somata were measured and averaged. The MdMNs were already enlarged before the presoldier molt, initially decreasing in size, before attaining the maximum size after differentiation. The different letters on the error bars indicate significant differences among stages/castes (one-way ANOVA followed by the Tukey-Kramer test, p<0.05).

To determine when the MdMNs become enlarged during soldier differentiation, backfill staining using fluorescent dye at several stages during differentiation was performed. We used the artificial induction method of soldier differentiation by JHA (juvenile hormone analog; pyriproxyphen) in *H. sjostedti* according to Ogino et al. [21]. Two stages were focused on during the differentiation process: “whitening pseudergates”, which were pseudergates with abdomens that had turned white 2 weeks after the application of JHA due to the gut purge preceding presoldier-molting, and “presoldiers”, which were 24 hours after presoldier-molting. The size of the MdMN somata in whitening pseudergates and presoldiers were 492.8±146.4 µm^2^ and 398.5±119.7 µm^2^, respectively (Fig. 4), indicating that the MdMNs became enlarged before presoldier molting and they then decreased in size after molting. The MdMNs then increase in size and attain their maximum size in the mature soldiers. These results for *H. sjostedti* show two important characteristics: (I) neuronal modifications are initiated before presoldier molting and, (II) the modification of the nervous system in soldier differentiation can be induced by JHA application as well as morphological changes associated with molting.

### Comparison of MdMNs in termites from other families

**Figure 5:**
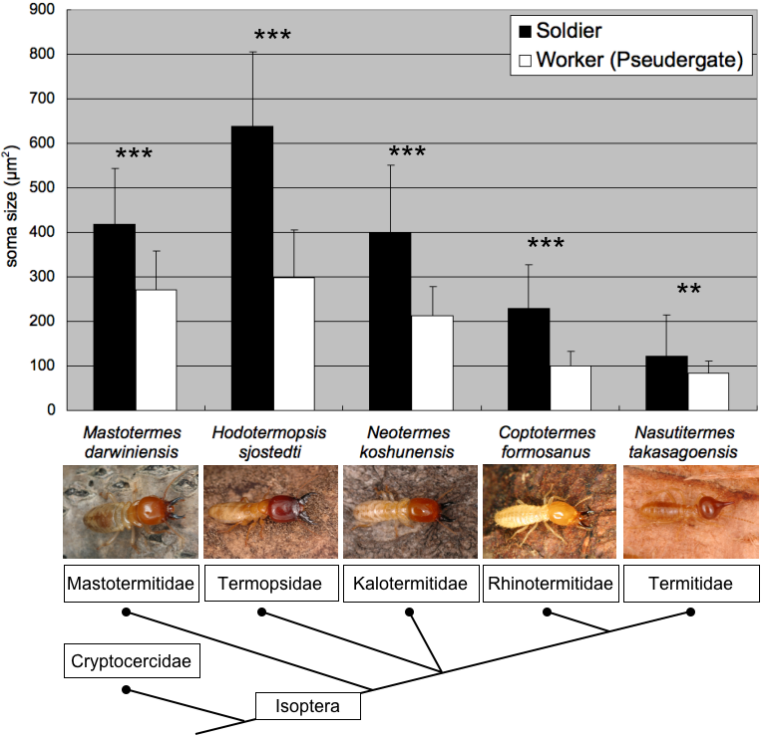
The size comparisons between soldiers and workers in other species belonging to various termite families (n = 4 for each caste in *Coptotermes formosanus* and n = 5 for each caste in other termite species). The areas of MdMN somata were measured and averaged. The enlargement of MdMNs was generally recognized in all termite lineages. The size differences between soldiers and workers (pseudergates) were significant in all of the species examined (**p<0.005 and ***p<0.001, Student's T-test). The dendrogram of termites is based on Eggleton et al. [37].

**Figure 6:**
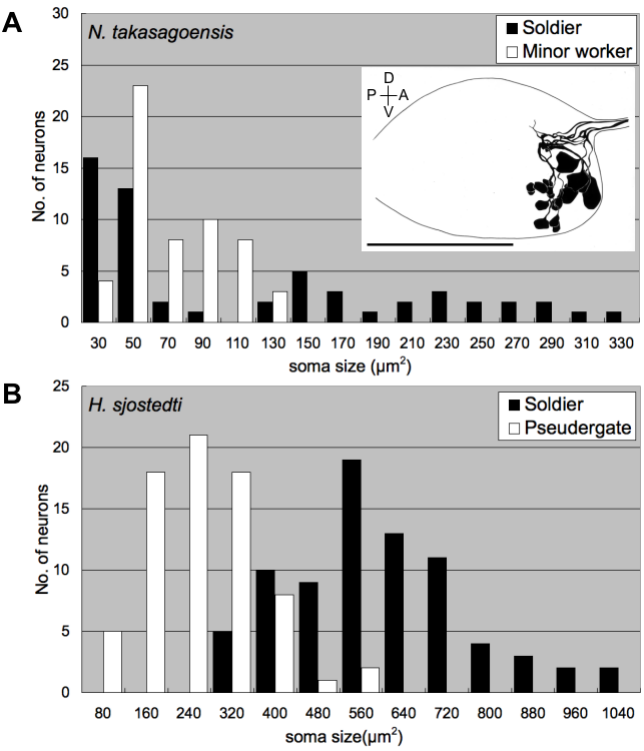
The size distributions of MdMNs in *N. takasagoensis* (A) and *H. sjostedti* (B). While the MdMNs exhibited a unimodal distribution in minor workers of *N. takasagoensis* (A) and in soldiers and pseudergates of *H. sjostedti* (B), the MdMNs in *N. takasagoensis* soldiers exhibited a bimodal distribution (A), indicating there are two size groups. Inset (A) shows a schematic lateral image of MdMNs in an *N. takasagoensis* soldier. Scale bar represents 100 µm.

To examine whether the enlargement of MdMNs in soldiers is a common phenomenon shared with other termite species, retrograde tracing of the MdMNs in soldiers and workers in other termite species was also performed. These termite families included *Mastotermes darwiniensis* (Mastotermitidae), *Coptotermes formosanus* (Rhinotermitidae), *Neotermes koshunensis* (Kalotermitidae) and *Nasutitermes takasagoensis* (Termitidae). Those MdMNs that exhibited characteristics similar to *H. sjostedti* were stained and the somata of these soldiers were also generally found to be larger than those of workers (Fig. 5). *Nasutitermes* soldiers, which have reduced mandibles and attack with frontal glands, had also several enlarged neurons. In nasute soldiers, interestingly, smaller neurons than those of minor workers, which can differentiate into nasute soldiers, were also stained (Fig. 6A). The MdMNs of *N. takasagoensis* soldiers could be divided into two groups based on their size, whereas the MdMNs of *H. sjostedti* and *N. takasagoensis* workers had unimodal size distributions (Fig. 6A, B). At least five enlarged neurons were recognized in *N. takasagoensis* soldiers, all of which were positioned anterior to smaller neurons (Fig. 6A, inset). The enlargement of MdMNs during soldier differentiation was demonstrated to be a common phenomenon over the termite lineages examined.

## Discussion

Caste in social insects is a special case of polyphenism in which colony members with similar (sometimes identical) genetic background have different morphologies and behaviors [22]. This polyphenic differentiation of caste contributes to the task allocation that increases the inclusive fitness of social insect colonies [1], [23], [24]. In order to understand the evolution of social insects, it is important to elucidate the proximate mechanisms of behavioral differentiation that are responsible for the development of elaborate social behavior [1], [15], [25]. Termites are hemimetabolous social insects with soldiers, workers (pseudergates) and alates. Comparisons of the nervous systems of soldiers and workers showed that soldiers have larger SOGs which process sensory information from the mouthparts and control their motion. The MdMNs in the SOG were observed to become enlarged during soldier differentiation and this feature was common to all of the termite lineages examined. The present study is the first report of neural modification in social insects, including social hymenopterans, in which motor neurons are morphologically altered during caste differentiation.

The soldiers of *H. sjostedti* have a well defined defensive behavior that involves turning their head to face intruders or predators, opening their mandibles, and then closing them quickly, while moving their bodies forward then backward [2]. The termite soldiers have markedly larger heads than pseudergates [26], which implies that soldiers have more developed mandibular muscles than pseudergates. When soldiers attack intruders, these developed muscles (most of which are closer muscles) will be contracted repeatedly through the action of neurotransmitters, which are diffused from synaptic vesicles found at the end plates of motor neurons [27]. This suggests that the relatively thicker muscles of soldiers require relatively more neurotransmitters. Consequently, the MdMNs of soldiers with their larger somata would likely produce more neurotransmitters for the contraction of mandibular muscles. Given that soldiers have thicker nerve roots innervating the mandibular muscles compared to pseudergates (Fig. 1F), and because neurons with large somata tend to have large axons [28], the MdMNs of soldiers should be concurrent with thick axons. Larger axon trunks tend to increase conduction velocity because of the decrease in electric resistance [27], and several examples have been reported in which neurons with thick axons have high pulse conduction [29], [30]. These neurons elicit fast and strong muscle movements, such as crayfish motor giant neurons (MoGs) that elicit tail-flip escape behavior. Similarly, the enlargement of MdMNs contributes to fast and strong mandibular movements of soldier defense behavior. We uniquely demonstrated that the MdMNs of soldiers and pseudergates elicit the contraction of developmentally identical muscles, however, only MdMNs of soldiers exhibit an increase in size and morphological changes during postembryonic development.

Furthermore, the developmental significance of MdMN enlargement may be considerable; MdMN enlargement occurs prior to the presoldier molt and it is possible that the larger neurons play a functional role in soldier differentiation. In the case of metamorphosis of holometabolous insects, it is known that the reconstruction of adult muscles (cf. thoracic muscles) requires the presence of certain motor neuron secretions innervating the muscles [31]. Similarly, in the course of soldier differentiation in termites, the muscles in the head capsule are first degenerated before presoldier molt and then reconstructed to produce thicker muscles after soldier molt (data not shown). Therefore, there is a possibility that the enlargement of MdMNs contributes to muscle reconstruction during the morphogenesis associated with soldier differentiation.

Koshikawa et al. showed that the application of JHA induced soldier-specific morphogenesis, such as the enlargement of head and mandibles in *H. sjostedti* [26], and extensive research has been conducted to date to show that JHAs induce soldier morphs in both lower and higher termite species [32]. Furthermore, their findings suggest that a high JH titer is effective for soldier differentiation [32]. The present study revealed that the application of JHA induced the enlargement of MdMNs indicating and that neuronal modification is also likely to be associated with a high JH titer. There are two possible explanations of the direct or indirect relationships: JH affects MdMNs hormonally, or due to other mediators like the muscle growth. The fact that neuronal enlargement preceded presoldier molting suggests that a high JH titer affects the relatively early stages of nervous modification as well as morphogenesis. Associated with soldier morphogenesis, behavioral changes may also be induced by JHA application. Previous studies have demonstrated that JH titer is involved in modulating various insect nervous systems (cf. in *Drosophila* [33], [34] and *Apis melifera* [35]). Similarly, in termites, a high JH titer may also modify other neuronal systems involved with aggressiveness or decision-making.

Given that all Isopteran families have soldiers, the soldier caste is considered to be an acquired condition of the common termite ancestor [36], [37]. In this study, the observed enlargement of MdMNs in soldiers was conserved in the representative species of all the major isopteran families examined, indicating that evidence of JH-dependent MdMN enlargement is likely to be important for soldier differentiation. This finding also suggests that, in addition to soldier aggression, this soldier-specific MdMN enlargement was only acquired once in the common ancestor of the taxa examined. Interestingly, the enlargement of motor neurons was not only observed in mandibulate soldiers, but also in nasute soldiers, which attack enemies with substances secreted from their frontal glands. It is known that nasute soldiers use their mandibular closer muscles to contract the frontal glands when they discharge defense secretion [38]. As in this report, the MdMNs of nasute soldiers occur as both partly enlarged and reduced in size. The partly enlarged MdMNs are thought to control the contraction of the frontal gland during defensive behavior and the latter may be used to close their small mandibles, although this still needs to be demonstrated. The finding that MdMNs of minor workers had MdMNs of intermediate size suggests that the nasute soldiers have enlarged neurons associated with defense behavior but degenerate MdMNs for the other neurons. In conclusion, despite the variety morphs and defensive mechanisms, the enlarged MdMNs are conserved in all termite species and are considered to be important for soldier evolution.

## Materials and Methods

### Insects

*Hodotermopsis sjostedti* (Family Termopsidae) is distributed throughout the Satsunan Islands of the Kagoshima Prefecture in southern Japan [39], [40]. Colonies were sampled from rotten wood in primary evergreen forests on Yakushima Island, Kagoshima Prefecture, in May 2004–2006. Colonies were kept in the laboratory as stock at approximately 25°C under constant darkness. To compare different species within the termite order (Isoptera), the following termite species from various families were collected: *Mastotermes darwiniensis* (Mastotermitidae) from Queensland, Australia, *Neotermes koshunensis* (Kalotermitidae) from Okinawa Prefecture, Japan, *Coptotermes formosanus* (Rhinotermitidae) from Tokyo, Japan, *Nasutitermes takasagoensis* (Termitidae) from Iriomote Island, Okinawa Prefecture. Japan. In *M. darwiniensis, N. koshunensis* and *C. formosanus,* normal workers and soldiers were examined. In *N. takasagoensis*, soldiers and minor workers were used. Minor workers of *N. takasagoensis* can differentiate into soldiers.

### Morphometric Study

To clarify the morphological differences in the CNS of *H. sjostedti* soldiers and pseudergates, the size of the brain (cerebral ganglion) and SOG, as well as the thickness of the mandibular and maxillary nerves were measured (N = 10, for each caste). Insects were anesthetized on ice, and the brain and SOG were removed from the head capsules by dissection in phosphate buffer saline (PBS), where after they were fixed in FAA fixative (formalin: acetic acid: ethanol = 6∶1∶16) and then transferred to 70% ethanol. The sizes (height and width) of the brain and SOG were measured using an image analysis system with a CCD camera (HIM-1, HOGA, Kyoto, Japan). The SOG length was defined as the distance from the rostral to the distal edges of the ganglion, and SOG width as the distance along the transversal axis between both edges of the ganglion. The brain width was defined as the distance between basal parts of both optic nerves of the brain, and the brain length as the distance between rostral-end of the brain to the basal part of the antennal lobe. The sizes of SOG and brain were compared using these parameters.

### Neuroanatomy

To compare the sizes of MdMNs, retrograde staining using fluorescent dye from mandibular muscles was performed [41]. In *H. sjostedti*, we used 5 specimens for each caste and measured 74, 74, 75 and 78 stained neurons (summation of 5 individuals) in pseudergate, whitening pseudergate, presoldier and soldier, respectively. The maxillary motor neurons were also stained and 5 neurons that were positioned on the lateral side of SOG and which stained consistently were also measured. For comparisons among termite species, 4 individuals (for each caste) of *C. formosanus* and 5 individuals for each caste of *M. darwiniensis*, *N. koshunensis* and *N. takasagoensis* were also examined. The termites were anesthetized on ice. To stain motor neurons innervating mandible closer muscles equally, the right hemisphere of the head capsule was cut transversely at half of head length with a sharp scalpel, and a small crystal of a fluorescent dextran (Fluororuby; Molecular Probes, Carlsbad, USA) was placed onto the surgically treated region. The fluorescent tracer was transported retrogradedly through the neurons of the CNS over 20–24 hours in relatively larger termites (*H. sjostedti* and *M. darwiniensis*), or 4–12 hours in the relatively smaller termites (*N. koshunensis, C. formosanus* and *N. takasagoensis*). The dissected termites were placed in Petri dishes lined with wet filter paper. After several hours, termites were decapitated and their heads were fixed in 4% paraformaldehyde overnight at 4°C and dehydrated using an ethanol series (70%, 90%, 100% for 10 min each). Samples were cleared with methyl salicylate before being observed with a confocal laser scanning microscope (FLUOVIEW FV1000 and FV300, OLYMPUS, Tokyo, Japan). The confocal images of each preparation were captured using a 1.0 µm stack in *N. koshunensis, C. formosanus* and *N. takasagoensis* and a 1.5 µm stack in *H. sjostedti* and *M. darwiniensis.* Images were stored as TIFF files for the somata size measurement. The section containing the most soma for each MdMN was removed for analysis using Image J software (http://rsb.info.nih.gov/ij/), and the soma area were averaged.

### Treatment with Juvenile Hormone Analogue

To observe the modification processes of the nervous system in the course of soldier differentiation, presoldiers were artificially induced by JHA treatment. In order to induce molting from pseudergates to presoldiers, pyriproxyfen (Sigma-Aldrich, St. Louis, USA) was used as a JHA. The experimental procedure employed for JHA application to termites was the same as that described in [21], in which presoldiers were efficiently induced in *H. sjoestedti*. The JHA pyriproxyfen, diluted in acetone, was aliquoted into filter paper-lined Petri dish with 70 mm diameter at a final concentration of 10 µg/dish. After evaporating the acetone, the filter paper was moistened with distilled water and 10 pseudergates of *H. sjostedti* were placed in each dish. Control dishes without JHA were also prepared. Filter papers with JHA were replaced after one week. Whitening pseudergates, identified by a whitening of the abdomen due to a gut purge before presoldier molting [21], and presoldiers within 24 hours after molting, were examined by retrograde staining using a fluorescent dextran.
